# New methods for sorghum transformation in temperate climates

**DOI:** 10.1093/aobpla/plad030

**Published:** 2023-06-03

**Authors:** Sara Miller, Asta Rønager, Rose Holm, Juan B Fontanet-Manzaneque, Ana I Caño-Delgado, Nanna Bjarnholt

**Affiliations:** Section for Plant Biochemistry, Department of Plant and Environmental Sciences, University of Copenhagen, Thorvaldsensvej 40, 1871 Frederiksbergs, Denmark; Copenhagen Plant Science Center, Department of Plant and Environmental Sciences, University of Copenhagen, Thorvaldsensvej 40, 1871 Frederiksberg, Denmark; Section for Plant Biochemistry, Department of Plant and Environmental Sciences, University of Copenhagen, Thorvaldsensvej 40, 1871 Frederiksbergs, Denmark; Copenhagen Plant Science Center, Department of Plant and Environmental Sciences, University of Copenhagen, Thorvaldsensvej 40, 1871 Frederiksberg, Denmark; Section for Plant Biochemistry, Department of Plant and Environmental Sciences, University of Copenhagen, Thorvaldsensvej 40, 1871 Frederiksbergs, Denmark; Copenhagen Plant Science Center, Department of Plant and Environmental Sciences, University of Copenhagen, Thorvaldsensvej 40, 1871 Frederiksberg, Denmark; Department of Molecular Genetics, Centre for Research in Agricultural Genomics (CRAG), CSIC-IRTA-UAB-UB, Campus UAB (Cerdanyola del Vallès), 08193 Barcelona, Spain; Department of Molecular Genetics, Centre for Research in Agricultural Genomics (CRAG), CSIC-IRTA-UAB-UB, Campus UAB (Cerdanyola del Vallès), 08193 Barcelona, Spain; Section for Plant Biochemistry, Department of Plant and Environmental Sciences, University of Copenhagen, Thorvaldsensvej 40, 1871 Frederiksbergs, Denmark; Copenhagen Plant Science Center, Department of Plant and Environmental Sciences, University of Copenhagen, Thorvaldsensvej 40, 1871 Frederiksberg, Denmark

**Keywords:** Agrobacterium, dhurrinase, sorghum, transformation, transient, tissue culture

## Abstract

Sorghum (*Sorghum bicolor*) is an emerging cereal crop in temperate climates due to its high drought tolerance and other valuable traits. Genetic transformation is an important tool for the improvement of cereals. However, sorghum is recalcitrant to genetic transformation which is almost only successful in warmer climates. Here, we test the application of two new techniques for sorghum transformation in temperate climates, namely transient transformation by *Agrobacterium tumefaciens–*mediated agroinfiltration and stable transformation using gold particle bombardment and leaf whorls as explants. We optimized the transient transformation method, including post-infiltration incubation of plants in the dark and using *Agrobacterium* grown on plates with a high cell density (OD_600_ = 2.0). Expression of the green fluorescence protein (GFP)-tagged endogenous sorghum gene *Sb*DHR2 was achieved with low transformation efficiency, and our results point out a potential weakness in using this approach for localization studies. Furthermore, we succeeded in the production of callus and somatic embryos from leaf whorls, although no genetic transformation was accomplished with this method. Both methods show potential, even if they seem to be influenced by climatic conditions and therefore need further optimization to be applied routinely in temperate climates.

## Teaser

Sorghum is a subtropical cereal crop with many good qualities such as nutritional value and high drought tolerance, a very valuable characteristic in the face of warming climate. Nevertheless, sorghum is considered under-utilized and not as much breeding progress has been made as in other major cereals. Knowledge of genes behind specific traits can considerably speed up plant breeding and help utilize sorghum's full potential. Genetic modification is a valuable tool for discovering gene functions, but genetic transformation is notoriously difficult in sorghum. The currently available methods are highly climate dependent and only successful in a handful of geographical locations. In this work, we test two new methods for sorghum transformation for their robustness in colder climates, show their potential, and implement and discuss their improvement.

## Introduction

Sorghum (*Sorghum bicolor*) is a hardy C_4_ cereal crop, especially valued for its high drought tolerance (Olson *et al.* 2012; Rooney 2014). Despite being the fifth most produced cereal worldwide (FAOSTAT 2022), it is considered an under-utilized crop and sorghum improvement lags behind other major cereals (O’Kennedy *et al.* 2006). Biotechnological approaches have been very successful in other cereals, especially maize, but sorghum is very recalcitrant to genetic transformation (Williams *et al.* 2004; O’Kennedy *et al.* 2006; Raghuwanshi and Birch 2010). Sorghum transformation is considered to mainly be possible in warmer climates since the growth conditions for the plants serving as donors for the explants for transformation, the immature embryos, are crucial for success (Zhao *et al.* 2000; Belide *et al.* 2017; Wang *et al.* 2021). Climate has been identified as one of the main conditions influencing transformation efficiency, and Belide *et al.* (2017) suggested an approach to use differentiating callus sourced from sorghum grown in summer months and maintained through winter for year-round transformation. However, even collaborators working in climates more suitable for sorghum report season-dependent transformation success rates due to unknown factors presumably related to sunlight (Godwin 2022). This challenges potential genetic studies of sorghum in temperate climates such as most regions of Europe where the interest in sorghum is otherwise increasing due to its high drought tolerance and other advantageous traits, making it a promising crop for future climates (Berenji and Dahlberg 2004).

Recently, two new methods for sorghum transformation were published. The first method is stable transformation modelled on the predominant method used for sugarcane (*Saccharum* ssp.; Silva *et al.* 2020), one of the closest relatives to sorghum (Saski *et al.* 2007; Cahoon *et al.* 2010). Sorghum is used as a genetic model to study sugarcane (Jannoo *et al.* 2007; Rokhsar *et al.* 2010) and was the key to annotating the complex sugarcane genome (Garsmeur *et al.* 2018). Because many sugarcane varieties are hybrids and do not produce seeds, the preferred explant in sugarcane transformation is leaf whorls, as frequently reported (Kumar *et al.* 2014; Wu *et al.* 2015; Zhang *et al.* 2015; Gao *et al.* 2016; Jung and Altpeter 2016; Sandhu *et al.* 2016). Sectioned or fragmented leaf whorls have been successfully used as explants in maize and sorghum transformation. This system is quite robust using an explant that is available all year. Silva *et al.* (2020) have already demonstrated that it is possible to use leaf whorls as explants for sorghum transformation, and with some improvements, the method could be standard in areas where immature embryo production is difficult.

The second method is transient transformation via agroinfiltration similar to the standard practice for *Nicotiana benthamiana* (Sharma *et al.* 2020). Agroinfiltration using *Agrobacterium tumefaciens* is a simple and fast method to study gene function that has been used in *N. benthamiana* for many years (Grimsley *et al.* 1986; Chen *et al.* 2013), and has also been adapted for several other species (Hoshikawa *et al.* 2019; Suzaki *et al.* 2019; Zhang *et al.* 2020). The *N. benthamiana* model system has also been useful for studying sorghum genes (Darbani *et al.* 2016; Pan *et al.* 2021), but as *N. benthamiana* is a dicot, it is far from the native system and therefore has its limits, especially when studying specialized metabolites that are frequently species specific. Another possibility for transient expression in monocots is protoplast transformation, but this approach also has several limitations. Protoplasts are cells without cell walls, which makes them delicate and difficult to handle and also quite distinct from a whole plant (Sharma *et al.* 2020). Additionally, only a few protoplast protocols for sorghum are available and the system is limited in regards to sub-cellular localization (Meng *et al.* 2020). Alternatively, engineered plant viruses have been used for transient expression, but the size of the DNA that is deliverable is limited and the virus can also affect plant development (Lee *et al.* 2015). Lastly, particle bombardment is used for transient expression in cereals (Takumi *et al.* 1994; Nielsen *et al.* 1999; Able *et al.* 2001), but it is relatively inefficient and laborious, and requires the preparation of large amounts of plasmid DNA as well as costly materials (Zhao and Tomes 2003; Xu *et al.* 2014). We, therefore, wanted to adapt both transient expression by agroinfiltration and stable transformation of leaf whorls as tools for sorghum transformation in temperate climates to study the function of genes from sorghum and other cereal crops and create knowledge to adapt sorghum to cooler climates.

Transgene expression in both transformation systems using the green fluorescence protein (GFP) as a reporter gene was tested, as well as expression of native sorghum genes in the transient expression system. Overall, the approaches are promising, but we encountered low transformation efficiency and issues with autofluorescence in both systems.

## Materials and Methods

### Regenerating sorghum from leaf whorls

#### Plant material and leaf whorl isolation.

Seeds from the sorghum lines BTx430 and BTx623 were planted in soil (peat soil with calcium, macro- and micro-nutrients [Urnjord]) in the greenhouse (25 °C, min. 16 h light, min 100 µmol m^−2^ s^−1^) with regular fertilizer application (10 % Pioner Basis Brun [NPK 14-2-23] + 0.04 % red iron oxide). Stems were harvested after 6–10 weeks once the plants had reached developmental Stage 3, just before the change from the vegetative into the reproductive stage, its end marked by flag leaf emergence. Stems were sterilized by spraying with 70 % (v/v) EtOH, the outer layers were removed aseptically and the sterilization with EtOH was repeated three times. Stems were cut into 2–3 mm thick slices producing 10–12 leaf whorls from each. The resulting leaf whorls were placed on 90 × 15 mm petri dishes with callus induction medium (CIM) and incubated in the dark at 28 °C.

#### Media for tissue culture.

A modified version of the media described by Liu and Godwin (2012) was used **[see****Supporting Information—Table S1****]**. The addition of α-lipoic acid (**[see****Supporting Information—Table S1**], Media 3, 4) to reduce the production of phenolic compounds and the substitution of sucrose for maltose (**[see****Supporting Information—Table S1****]**, Media 2, 4) to increase the formation of somatic embryos were tested as this had been shown to improve callus formation from sorghum before (Belide *et al.* 2017).

#### Callus induction and regeneration.

Two different morphogenic paths for regeneration were tested, direct somatic embryogenesis (DSE) without an intervening callus phase, and indirect somatic embryogenesis (ISE) where somatic embryos are generated from callus. For DSE, plants were transferred to regeneration medium (REM) and placed in light (100 µmol s^−1^m^−2^, 16 h light/8 h darkness, 28 °C) after 9 days on CIM. For ISE explants remained on CIM for 2 months being sub-cultured every 10 days. Necrotic tissue was removed at every sub-culturing step until only a compact globular callus was left. The callus from each petri dish was weighed to assess the amount of callus formed on different types of media and then transferred to REM and placed in light (100 µmol s^−1^m^−2^, 16 h light/8 h darkness, 27 °C). Shoots were transferred from SRM to rooting medium (RM) when 3–5 cm tall.

#### Transformation by particle bombardment.

For biolistic transformation, a plasmid containing eGFP driven by the *Zm*Ubi promoter (Lamont *et al.* 2017) was purified from 5 mL overnight culture of *E. coli***[see****Supporting Information—****Protocol S2]**. Gold particles were coated as described by Liu *et al.* (2014). Leaf whorls or leaf whorl-derived callus were used for transformation either 7 days (young leaf whorls), 15–18 days (old leaf whorls) or 2 months (callus) after callus induction. Explants were placed in the middle of a 90 × 15 mm petri dish with osmotic medium 3 h prior to bombardment. DNA-coated gold particles were delivered using a Biolistic PDS 1000/He Particle Delivery System (BIO-RAD). The distance from the microcarrier assembly to the explants was adjusted to either 5 or 8 cm and the helium pressure to 1100 kPa. Approximately 1.4 µg plasmid was loaded onto each macrocarrier, and 27 Hg vacuum was generated prior to each shot. Explants were kept on OSM for 3 h after bombardment, then transferred to CIM and kept in the dark at 28 °C. After 4 days, they were transferred to REM and placed in light (100 µmol s^−1^m^−2^, 16 h light/8 h darkness, 27 °C). From 4 days after bombardment, GFP expression was assessed regularly using a Leica M205 FA fluorescence stereo microscope.

#### Statistical analysis.

To determine which genotype and medium yielded the most callus, a two-way ANOVA was performed, using the software Graphpad 9 and Šidák’s multiple comparison test at 95% confidence.

### Sorghum agroinfiltration

#### Constructing expression vectors.

To increase the low transgene expression obtained by Sharma *et al.* (2020), a vector routinely used for transient expression in tobacco, pCAMBIA1300 (Nour-Eldin *et al.* 2006), was modified to be more suitable for transgene expression in sorghum by replacing the CaMV 35S promoter **[see****Supporting Information—****Table S3 and Figure S4]**, which is not very efficient for monocot transformation (Li *et al.* 2014). Two promoters used in stable transformation *Sb*U6-2 and *Sb*U6-3 (Massel *et al.* 2021) were amplified from sorghum gDNA attaching restriction sites, and the *Zm*Ubi promoter from plasmid DNA (pUKN) received from Liu and Godwin (2012) attaching Gibson overhangs. *Sb*U6-2 and *Sb*U6-3 were inserted into pCAMBIA1300 by restriction cloning whereas *Zm*Ubi was inserted via Gibson Assembly using Gibson Assembly Master Mix (NEB) and the correct integration verified by Sanger sequencing (Macrogen Europe).

#### Inserting coding sequences into the constructed expression vectors.

The maize codon-optimized eGFP was obtained from synthetic DNA (TWIST Biosciences) **[see****Supporting Information—****Figure S5]**. The coding sequence of *Sb*DHR2 was cloned from cDNA extracted from roots of 3-day-old seedlings of BTx623 sorghum, whereas the coding sequence of p19 was obtained from a plasmid (Bassard *et al.* 2012). The genes were amplified as described for pCAMBIA1300 above and inserted into the expression vectors with USER cloning (Nour-Eldin *et al.* 2006).

#### Sub-cellular localization of SbDHR2.

For sub-cellular localization, the Gateway expression vector R4pGWB604 with a GFP tag at the C-terminus (Nakagawa *et al.* 2008) was used. For Gateway cloning, *Sb*DHR2 was amplified from the previously constructed expression plasmids attaching attB1 and attB2 flanking sites. The resulting entry clone (pDONR221 backbone) and an entry clone containing *Zm*Ubi (pDONR P4P1r backbone) available in our lab (Kim *et al.* 2013) were inserted into the destination vector in an LR reaction.

#### Sorghum agroinfiltration.

Sorghum plants from the lines BTx623 and BTx430 were grown in the greenhouse at 25–28 °C with a minimum of 16 h of light. Plants were used for infiltration after 2–3 weeks when they had reached the 3-leaf stage. *Agrobacterium tumefaciens* strain GV3101 was grown overnight at 28 °C in YEB medium containing the appropriate antibiotics. The *A. tumefaciens* was re-suspended in an infiltration buffer as described by Sharma *et al.* (2020) and incubated for 3 h at RT prior to infiltration. The infiltration was conducted as described by Sharma *et al.* (2020). GFP fluorescence was assessed with a FV1000 confocal laser scanning biological microscope (Olympus).

## Results

### Callus formation

**Figure 1.**
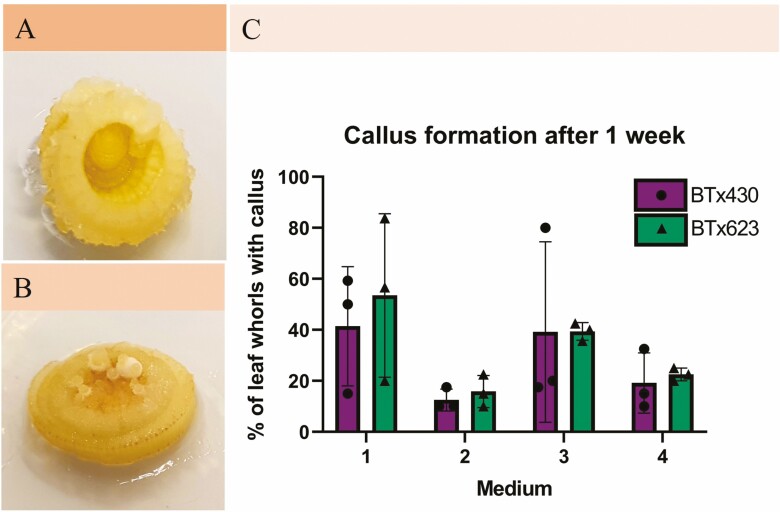
**Callus formation 1 week after induction**. The number of leaf whorls exhibiting callus formation after 1 week on CIM was counted to assess the difference between the different media and genotypes in the initial phase of callus formation. (A) Leaf whorl from the genotype BTx430 exhibiting callus formation at the cut edges. (B) Leaf whorl from the genotype BTx623 with somatic embryos beginning to form in the centre. (C) Callus formation was assessed in leaf whorls initiated in 3 consecutive weeks after they had been on CIM for 1 week and the number of leaf whorls showing callus formation counted. The graph shows the percentage of leaf whorls exhibiting callus formation.

While we initially had some success with callus formation, at some point, experiments no longer yielded any callus although several attempts were made at different times of year. Hence, some of the observations reported in the following cannot be supported by, for example, statistics as experiments could not be repeated, but may nonetheless be useful in planning strategies for the future development of sorghum tissue culture approaches. Callus formation was assessed on leaf whorls just before those destined for DSE were transferred to REM (Fig. 1). At this first assessment, it appeared like callus was most frequently produced from BTx623 on Medium 1. The two-way ANOVA revealed that only the difference between the different types of media was significant (P-value = 0.031) and that this accounted for 40.7 % of the variation showing that the substitution of sucrose for maltose is not beneficial for initial callus formation, as the two maltose containing media had the lowest callus formation. Likewise, the addition of the antioxidant α-lipoic acid did not have a positive effect on initial callus formation.

**Figure 2.**
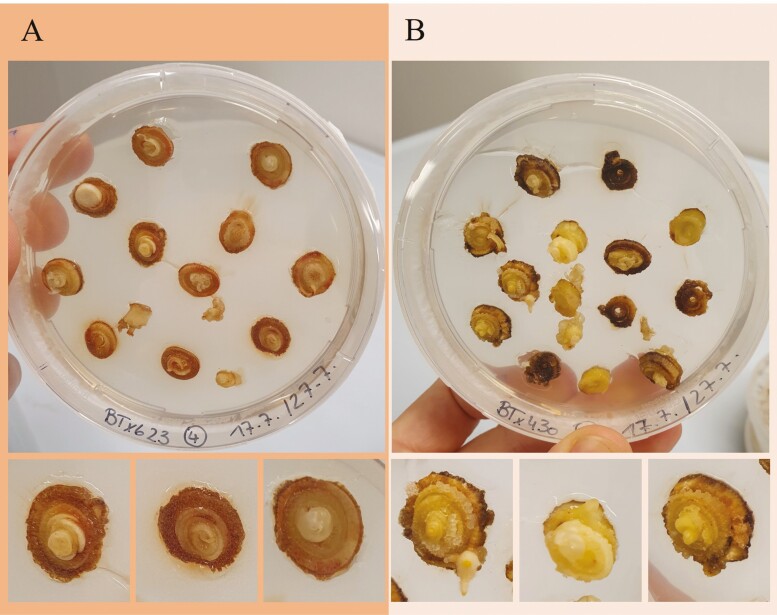
**Leaf whorls at 17 days after induction.** Varying degrees of browning were observed on all four types of CIM. This example shows a comparison of the two genotypes BT623 (A) and BTx430 (B) on CIM 4. Although both genotypes show a similar amount of browning. However, most BTx430 leaf whorls still produce white callus and somatic embryos whereas on BTx623 explants all the previously healthy callus has browned and died.

Although initially, both genotypes performed equally well, later in the callus initiation phase, BTx623 produced much more phenolics than BTx430, and next to no callus could be obtained from BTx623 (Fig. 2). Therefore, for all later experiments, only BTx430 was used to produce callus and to regenerate plants through ISE.

**Figure 3.**
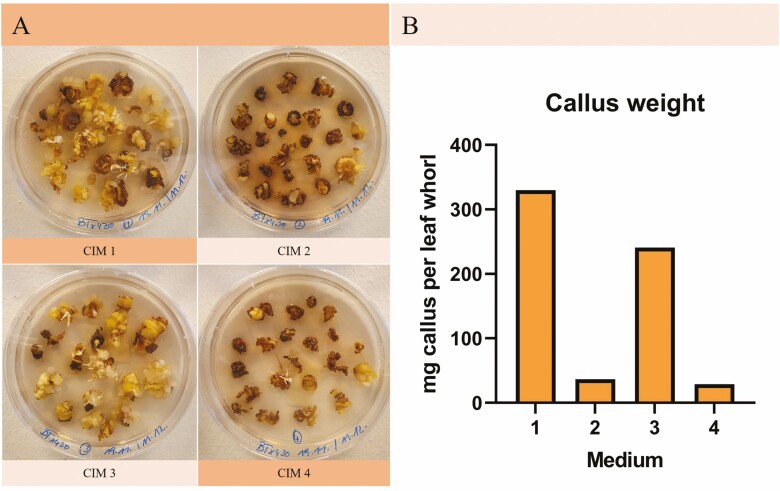
**Callus formation 30–60 days after induction.** (A) Callus on all four types of CIM 30 days after induction. CIM 1 represents the basic medium used successfully in previous studies for callus formation from immature embryos (Liu and Godwin 2012) and leaf whorls (Silva *et al.* 2020). In CIM 2, maltose is used as a carbon source instead of sucrose as this can aid the formation of somatic embryos (Gill *et al.* 2004; Belide *et al.* 2017). CIM 3 differs from the basic medium by the addition of α-lipoic acid which has the ability to reduce the production of phenolic compounds in tissue culture (Belide *et al.* 2017). CIM 4 represents a combination of the two previous media containing both maltose and α-lipoic acid. After a month, compact white callus had formed on CIM 1 and CIM 3 whereas most leaf whorls on CIM 2 and CIM 4 turned brown. (B) The callus was weighed 60 days after induction and the callus fresh weight (mg) per leaf whorl was calculated.

The trend observed during initial callus formation became even more pronounced as the experiment progressed and most of the explants on medium with maltose (CIM 2 and CIM 4) turned brown and died after about a month (Fig. 3A). This was also reflected in the amount of callus fresh weight produced per whorl (Fig. 3B), showing a clear difference between media with, respectively, maltose (CIM 2 and CIM 4) and sucrose (CIM 1 and CIM 3). No reduction in the formation of phenolics was observed upon the addition of α-lipoic acid; in fact, the opposite appeared to be the case, hence callus formation was higher without α-lipoic acid (CIM 1). As this experiment was only conducted in one replicate of 48 leaf whorls per type of medium, no statistical analysis was conducted. Any attempts to repeat the experiments did not yield any callus; however, the same trends were observed in preceding experiments where the amount of callus had not been weighed.

### Regeneration via DSE or ISE from leaf whorls

**Figure 4.**
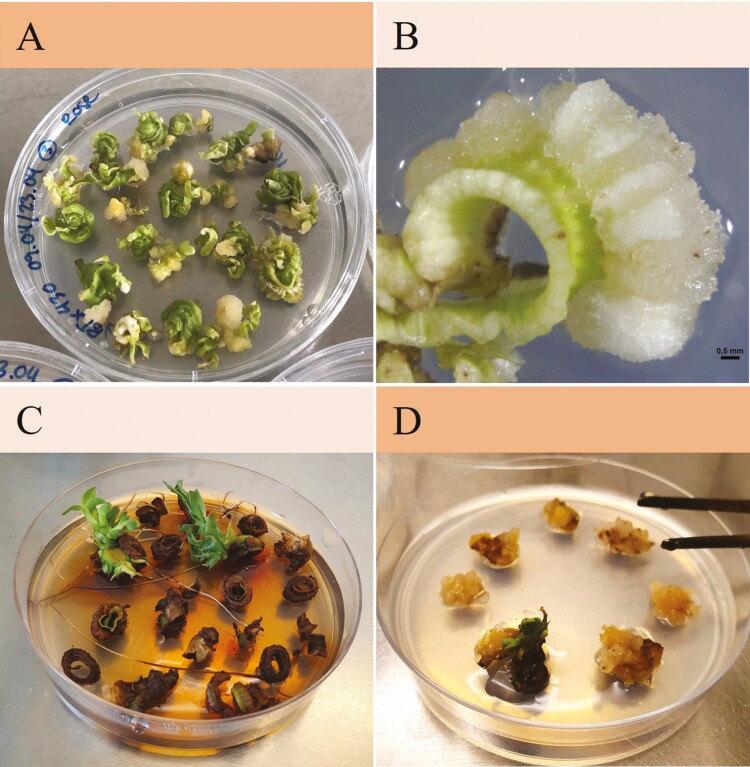
**Plants regenerating from leaf whorls.** Two regeneration paths were tested DSE where leaf whorls were transferred to REM 10 days after induction and ISE where leaf whorls were placed onto REM after 60 days once they had formed callus. With either methods, only very few plants could be regenerated. (A) Leaf whorls with compact white callus and somatic embryos turned green shortly after being transferred to REM. (B) Close-up of a leaf whorl on REM with a white compact callus and the original leaf whorl turned green. (C) Leaf whorls (BTx623) transferred onto REM after 10 days on CIM to achieve regeneration through DSE. (D) Callus (BTx430) transferred onto REM after 60 days meant to regenerate through ISE.

Explants destined for DSE turned green soon after being transferred to REM and started forming shoots after about 14 days on REM (4A). Shoots formed from BTx623 on REM 1 and 2, and from BTx430 on REM 2, 3 and 4. However, the growth of shoots depended less on genotype or medium, but rather on which part of the stem the leaf whorls originated from. Shoots only grew from whorls that were cut close to the base of the stem (Fig. 4) and no somatic embryos developed into shoots. It is, therefore, likely that the shoots regenerating were axillary buds sprouting, something that can also be achieved without going through the laborious tissue culture process (Rea and Karper 1932). After this discovery, regenerating leaf whorls through DSE was not attempted anymore.

The callus transferred to REM did not turn green nor did it form shoots. Only in the instances where remaining tissue from the original leaf whorl explants was left, a colour change was observed and in one case a shoot formed which was most likely from an axillary bud (Fig 4B).

### GFP expression in leaf whorls and leaf whorl-derived callus

**Figure 5.**
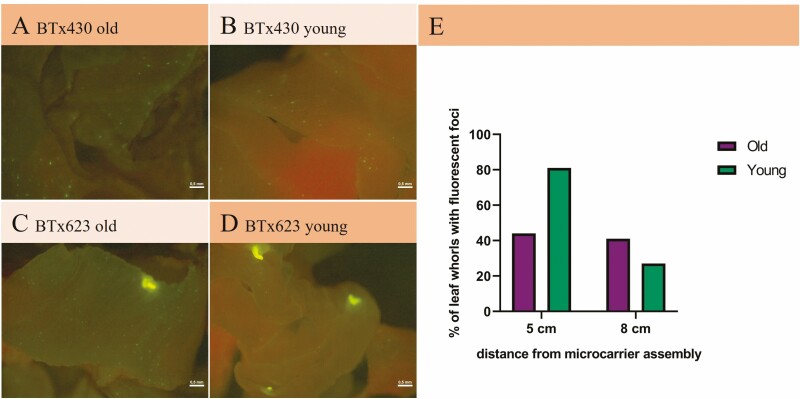
**Fluorescence in leaf whorls after particle bombardment.** Leaf whorls were bombarded for 7 (young) or 15–18 (old) days with gold particles coated with a plasmid containing eGFP (Lamont *et al.* 2017). For bombardment, the explants were placed either 5 or 8 cm from the microcarrier assembly. (A–D) Leaf whorls exhibiting a number of small and a few larger fluorescent foci. (E) The percentage of leaf whorls with fluorescent foci 4 days after bombardment.

When bombarded samples were inspected under a fluorescence microscope with a GFP filter, a signal was detected in leaf whorls (Fig. 5A–D), but not leaf whorl-derived callus. BTx623 and BTx430 showed fluorescence equally frequently. The highest number of leaf whorls with fluorescent foci was achieved with young leaf whorls positioned 5 cm from the microcarrier assembly upon bombardment, whereas the distance did not seem to matter for old leaf whorls (Fig. 5E).

### Optimizing transgene expression through agroinfiltration

**Figure 6.**
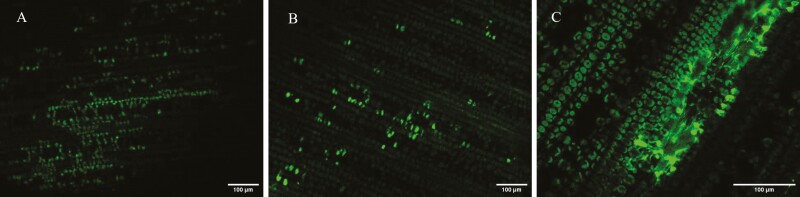
**Green fluorescence signal from sorghum leaves.** Green fluorescence was detected in sorghum leaves when replicating the transformation protocol described by Sharma *et al.* (2020). (A) Control that was only infiltrated with silencing suppressor p19 showing autofluorescence caused by tissue damage. (B and C) Leaf infiltrated with p19 + eGFP resulting in autofluorescence, but no GFP expression. (C) The area where the syringe was placed for infiltration, and wounding resulted in a particularly bright fluorescent signal.

In the first transformation attempts, the fluorescence signal was detected in leaves infiltrated with GFP as well as in the control only infiltrated with p19 (Fig. 6), no matter if GFP was driven by *Zm*Ubi, *Sb*U6-2, *Sb*U6-3 or CaMV 35S. None of the signals appeared to originate from GFP expression, which should be cytosolic and hence visible between the cell wall and the vacuole of the elongated epidermal cells, as well as in the nucleus due to diffusion. Optimization was, therefore, required to boost transgene expression and to distinguish true GFP signal from background signal coming from the wounding response from sorghum. To increase the virulence of *Agrobacterium*, 20 µM acetosyringone was added to overnight cultures of *Agrobacterium*, as this has previously been shown to be beneficial in stable and transient transformation of several different species (Rao and Rao 2007; Afolabi-Balogun *et al.* 2014; Zhang *et al.* 2020). To enable us to distinguish between autofluorescence from sorghum and GFP fluorescence, most of the optimization experiments were conducted expressing the GFP tagged *Sb*DHR2 driven by the *Zm*Ubi as this promoter has been shown to be the most effective in stable and transient expression of sorghum (Liu and Godwin 2012; Sharma *et al.* 2020). The sequence of DHR2 contains a putative N-terminal plastid signal peptide (Cicek and Esen 1998) which was supported by analysis of the sequence in TargetP 2.0 (Emanuelsson *et al.* 2007). We, therefore, expected to find expression of DHR2-GFP in the plastids.

Several different conditions to increase transgene expression were tested: the plants were placed in darkness at RT for 24 h after infiltration before being transferred back into the greenhouse (Zhang *et al.* 2020). GFP expression was assessed over the course of 2–6 days after infiltration (Leuzinger *et al.* 2013; Norkunas *et al.* 2018). Growing *Agrobacterium* on plates for 2 days rather than in liquid culture overnight was tested (Zhang *et al.* 2020). The influence of the cell density of the *Agrobacterium* culture on transgene expression was also tested using OD_600_’s of 0.2, 0.5, 1.0 and 2.0 (Norkunas *et al.* 2018). Lastly, another *Agrobacterium* strain was tested. The strain GV3101 was used to develop the sorghum agroinfiltration system by Sharma *et al.* (2020) and it is used for sorghum stable transformation as frequently as AGL1 (Elkonin *et al.* 2017; Wu and Zhao 2017; Do *et al.* 2018), the traditional strain for infiltration of *N. benthamiana* (Norkunas *et al.* 2018). On the other hand, EHA105 is often used for stable transformation of rice and maize (Kim *et al.* 2009; Pandey *et al.* 2010), and this strain was therefore selected for testing in sorghum agroinfiltration.

**Figure 7.**
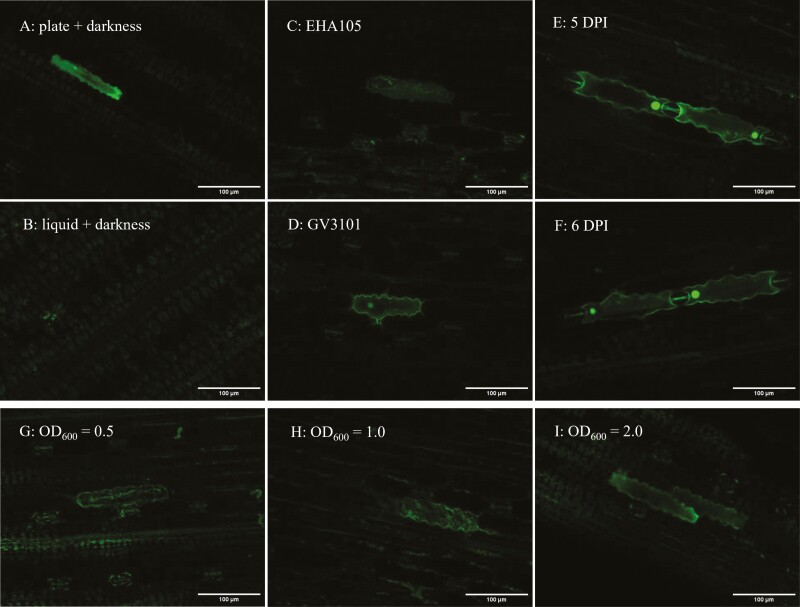
**Testing different conditions for transient transformation expressing DHR2-GFP.** To increase the transgene expression several adjustments to the originally published protocol (Sharma *et al*., 2020) were made. Growing Agrobacterium on plates, using higher Agrobacterium cell densities, incubating the plants in the dark after infiltration and inspecting plants 5 DPI improved transgene expression, whereas using the Agrobacterium strain EHA105 instead of GV3101 did not boost expression. (A and B) Expression of DHR2-GFP after incubation of the plants in the dark and with Agrobacterium grown on plates (A) or from liquid culture (B). (C and D) Expression of DHR2-GFP with Agrobacterium strains EHA105 (C) and GV3101 (D). (E and F) Expression of DHR2-GFP at 5 DPI (E) and 6 DPI (F). (G–I) DHR2-GFP expression using different cell densities, OD_600_ = 0.5 (G); 1.0 (H) or 2.0 (I).

**Figure 8.**
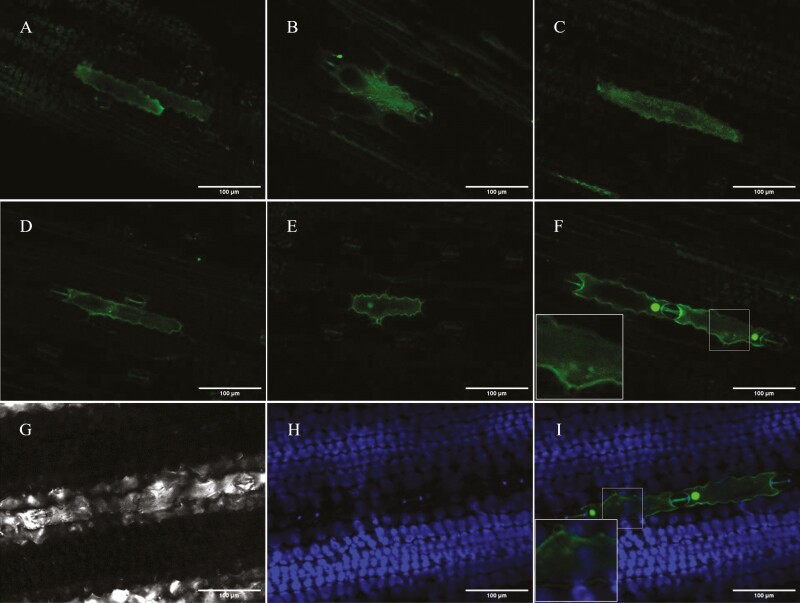
**Sub-cellular localization of SbDHR2.** Expression of SbDHR2 was observed in the cytosol (A–F), in the nucleus (D–F) and possibly in plastids (B, C, E and F). To determine if GFP fluorescence localized in the plastids, it was overlaid with chlorophyll autofluorescence (G–I): (G) bright field image (H) chlorophyll fluorescence (I) overlay of GFP and chlorophyll fluorescence. Images were taken 5 (A–F) or 6 DPI (G–I).

Incubation in the dark did not result in DHR2-GFP expression at first but when combined with the use of *Agrobacterium* grown on plates a cytosolic signal for *Sb*DHR2-GFP could be detected (Fig. 8A), whereas no signal was detected using *Agrobacterium* from liquid culture (Fig. 8B). The different cell densities were tested at the same time when the time course was conducted. No signal could be detected at any cell density 2, 3 or 4 DPI **[****Supporting Information—Figure S6****]**. Five DPI expression of *Sb*DHR2-GFP could be observed with an OD_600_ of 0.5 or higher (Fig. 7H and I). The highest expression could be detected 5 DPI using an OD_600_ of 2.0 (Fig. 7K). Expression persisted until 6 DPI, although it faded slightly (Fig. 7F). When comparing the two *Agrobacterium* strains, GV3101 yielded higher expression than EHA105 (Fig. 7E and F). Importantly, the signal was now detected in a pattern consistent with cytosolic expression. In summary, the best results were achieved when *Agrobacterium* was grown on plates, diluted to an OD_600_ of 2.0, the plants were incubated in the dark at RT 24 h post-infiltration and the GFP signal was assessed 5 DPI. Expression varied substantially between transformation events and was generally relatively low compared to what can be achieved in other plants. Whereas during springtime, transgene expression was achieved to a varying degree, but consistently, no transgene expression was observed during experiments conducted in summer.

### Sub-cellular localization of *Sb*DHR2

Contrary to the predictions made about the localisation of *Sb*DHR2, the gene was mainly found to be localized in the cytosol, and when very high expression was achieved also in the nucleus (Fig. 8). However, in some cases granular accumulation of GFP signal could be observed that did not align with chlorophyll fluorescence (Fig. 8I) but could constitute a localization in other plastid types without chlorophyll. This granular accumulation is especially visible in Figure 8F.

## Discussion

### Callus formation from leaf whorls

In this study, we tested the potential of leaf whorls as explants in sorghum transformation in colder climates. We achieved callus formation almost all year round even when the plants were grown in a semi-controlled environment in the temperate climate of Denmark where sorghum does not thrive outdoors. This indicates that leaf whorls could be a more robust explant in comparison to the traditionally used immature embryos that cannot be produced in sufficient quality all year round even in climates more suited for growing sorghum (Belide *et al.* 2017). Additionally, leaf whorls are easier to isolate than immature embryos and plants do not need to be grown until the reproductive stage, reducing the experimental time.

As well as testing an explant new to sorghum transformation, we also tested different media compositions. The addition of α-lipoic acid has previously resulted in increased production of embryogenic callus from immature embryos from sorghum (Belide *et al.* 2017) and has increased the transformation efficiency in several other crops among them another cereal, namely wheat (Dan *et al.* 2009). This positive effect of α-lipoic acid was not observed in this study, however. α-Lipoic acid did not lead to higher callus formation nor the increased formation of somatic embryos. Belide *et al.* (2017) reported that calli derived from immature sorghum embryos grown with α-lipoic acid looked different from calli grown without α-lipoic acid, displaying a more nodular structure and deeper green colour. We did not observe such a visual difference, as calli grown with or without α-lipoic acid both ranged in colour from white to yellow and were compact and globular. The addition of maltose likewise had no positive effect on the formation of callus or somatic embryos in our study. Maltose has previously increased the formation of somatic embryos from sugarcane leaf whorls (Gill *et al.* 2004), enhanced callus regeneration in wheat (Mendoza and Kaeppler 2002) and increased callus formation in rice (Xie *et al.* 1995; Binte Mostafiz and Wagiran 2018). Maltose has also been successfully used in the formation and maintenance of sorghum calli before (Belide *et al.* 2017; Dreger *et al.* 2019). Whereas in our study, α-lipoic acid slightly lowered callus formation, maltose had a very clear negative effect and almost no callus was formed as leaf whorls turned brown and died quickly on media containing maltose. This discrepancy might be explained by the different explants used to produce callus. Belide *et al.* (2017) used immature embryos while Dreger *et al.* (2019) used seedling shoot tips and also observed more callus formation in a medium with sucrose.

Lastly, we tested two different genotypes: BTx430 is well known to be more amenable to transformation and has been used in many studies (Nelson *et al.* 2011). BTx623 is a short-stature early maturing variety frequently used for generating hybrids for grain production and also serves as the sorghum reference genome (Cooper *et al.* 2019), and it is, therefore, attractive to be able to transform this cultivar. Even though initial callus formation from BTx623 was at least as high as from BTx430, BTx623 explants died after a couple of weeks on CIM. This was likely due to excessive production of phenolics as it was observed for leaf whorls from the genotype P898012 (Silva *et al.* 2020). In conclusion, the strategy for callus formation from sorghum leaf whorls presented by Silva *et al.* (2020) using BTx430 and the CIM developed by Liu *et al.* (2015) was the most successful in our study.

### Regeneration of plants from leaf whorls and leaf whorl-derived callus

While regeneration of shoots was difficult to achieve, our study indicated that axillary buds could be alternative explants to leaf whorls, as regeneration was only achieved from explants that presumably included axillary buds and not from the somatic embryos formed. Indicators to support this were that more plants regenerated from explants that were placed on REM 9 days after the start of callus induction when they were still intact leaf whorls, and only from leaf whorls originating from the lower part of the stem where axillary buds are expected to be found (Kebrom and Mullet 2015). Axillary buds have been successfully used for the transformation of maize (Sidorov *et al.* 2006) and sugarcane (Bower and Birch 1992; Gambley *et al.* 1993; Manickavasagam *et al.* 2004; Kumar *et al.* 2014; Mayavan *et al.* 2015), resulting in especially high transformation efficiencies when *Agrobacterium* was used to transform sugarcane (Manickavasagam *et al.* 2004; Kumar *et al.* 2014). The use of axillary buds might not only be more robust, but also bypasses the callus phase reducing somaclonal variation and speeding up the tissue culture process (Shrawat and Lörz 2006).

Although regeneration was achieved through neither DSE nor ISE, somatic embryos were observed in both treatments. Silva *et al.* (2020) employed ISE when transforming sorghum leaf whorls, but regeneration through DSE has been achieved from sugarcane leaf whorls many times (Huckett *et al.* 2000; Kalunke *et al.* 2009; Vyver 2010; Taparia *et al.* 2012a, 2012b; Kumar *et al.* 2014; Jung and Altpeter 2016). We used the same medium for ISE as well as DSE, containing the growth regulator 2,4-D that is commonly used for callus induction (Ball *et al.* 1993; Malik *et al.* 2003; Tahir *et al.* 2011) and has also been used for DSE from sugarcane leaf whorls (Huckett *et al.* 2000; Kalunke *et al.* 2009; Vyver 2010), but in lower concentrations than here. The majority of studies use NAA or a combination of NAA and kinetin for DSE embryogenesis from sugarcane (Desai *et al.* 2004; Taparia *et al.* 2012a, 2012b; Kumar *et al.* 2014; Jung and Altpeter 2016; Sandhu *et al.* 2016). Hence, adjusting growth regulators specifically to suit DSE may benefit the use of leaf whorls for transformation.

The failure of the regeneration through ISE, but also the fact that callus formation from leaf whorls was not achieved anymore after about one year of experiments might be attributed to the growing conditions of the plants in the greenhouse. Silva *et al.* (2020) reported that plants needed 30 days to reach developmental stage 3 whereas in our greenhouse that took at least 6 weeks and in winter up to 10 weeks. This indicates that our conditions were less optimal for sorghum growth. Liu *et al.* (2014) stress the importance of the right growth conditions for the plants when producing immature embryos, and that calcium deficiency or a pest infestation can be detrimental to the quality of immature embryos. It is therefore likely that the growing conditions for sorghum need to be further optimized to ensure continued production of callus and plant regeneration from leaf whorls. Application of additional calcium fertilizer is recommended (Liu *et al.* 2014), but increasing the growing temperature and light and with it, the transpiration rate could also help facilitate the movement of Ca^2+^ in the plant (Gilliham *et al.* 2011). As a C_4_ plant sorghum has a very high photosynthetic potential (Christin and Osborne 2014) and therefore thrives under high light intensities. These high light intensities can be difficult to provide in greenhouse facilities. While we aimed to implement a system allowing for sorghum transformation without requirement for highly optimised growth conditions or excessive use of space in growth cabinets, providing more controlled environmental conditions for the growing sorghum, might be necessary to achieve sorghum transformation in temperate climates. This would be possible with leaf whorls sourced from younger plants grown in growth chambers or *in vitro* as it has been done for maize before (Lowe *et al.* 2016; Jones *et al.* 2019) and very recently also sorghum (Wang *et al.* 2023)

### GFP expression in leaf whorls and leaf whorl-derived callus

A fluorescence signal was detected from leaf whorls but not from callus bombarded with DNA-coated gold particles. A possible explanation is that after 2 months on CIM, the callus was no longer viable enough to express transgenes. Although Silva *et al.* (2020) proliferated callus from leaf whorls for up to 60 days to assess the accumulation of phenolics, they used a 4-week-old callus for transformation. Likewise, Liu *et al.* (2015) found that callus from immature embryos lost its ability to regenerate after 4 weeks and that regeneration was highest after 2 weeks. Consequently, the 2-month-old callus used here may have had reduced viability and transformation and regeneration potential. It is also possible that the fluorescence, which was exclusively observed in leaf whorls was not GFP signal, but autofluorescence due to tissue damage as demonstrated for leaves in our agroinfiltration experiments. Experiments with younger callus as well as with controls bombarded with uncoated gold particles were planned, but at this point, callus formation from leaf whorls was not achieved regularly anymore.

### Optimizing transgene expression through agroinfiltration

While the initial goal was to explore transient gene expression in sorghum and possibly improve it by using endogenous sorghum promoters, the first experiments showed similar fluorescence patterns in controls and leaves infiltrated with constructs containing GFP (Fig. 6). Untreated leaves showed next to no fluorescence, only a row of fluorescent cells could be seen above the longitudinal vasculature of the leaf which can most likely be attributed to silica-accumulation (Dabney *et al.* 2016; Kumar *et al.* 2017). Grasses are well known for taking up silicic acid from soil and depositing it as silica in their cells (Kumar *et al.* 2017). These silicate inclusions serve as defense against various stresses and their formation increases upon wounding (Hall *et al.* 2019). One way to detect them is with the help of a GFP fluorescence filter (Dabney *et al.* 2016). It can, therefore, be assumed that the damage resulting from infiltration and/or the *Agrobacterium* infection induces fluorescence in sorghum leaves. The strongest signal was observed where the leaf was pricked and the syringe placed for infiltration (Fig. 6C), which supports the hypothesis that the fluorescence observed in initial experiments was mainly damage induced. We, therefore, explored experimental conditions that could potentially improve transgene expression and enable us to distinguish damage-induced fluorescence from GFP fluorescence.

Even though DHR2 did not localize where expected, using this GFP-tagged endogenous sorghum gene was an important aid in enabling us to distinguish GFP fluorescence from damage-induced fluorescence. The second finding contributing to the detection of increased transgene expression was that expression peaked 5 DPI (Fig. 8E). Sharma *et al.* (2020) inspected leaves at 3-4 DPI where we were almost always unable to detect any expression. This seems to be unusual as in most plants transgene expression is detected 2–3 DPI (Norkunas *et al.* 2018; Suzaki *et al.* 2019; Zhang *et al.* 2020) although some studies report a later expression peak depending on the *Agrobacterium* strain (Norkunas *et al.* 2018) or expression vector used (Leuzinger *et al.* 2013). The third key factor increasing transgene expression was the cell density of *Agrobacterium* used for infiltration. We achieved the best result with OD_600_ = 2.0, the highest density tested. This is also relatively unusual as in most other studies an OD_600_ of 0.5–1.0 is used (Yang *et al.* 2000; Vaghchhipawala *et al.* 2011; Ma *et al.* 2012; Sharma *et al.* 2020; Zhang *et al.* 2020). It has been shown before that particularly low cell densities result in significantly lower transgene expression in *N. benthamiana*, but increasing cell densities to above OD_600_ = 0.8 did not result in significantly higher transgene expression (Norkunas *et al.* 2018; Varchenko *et al.* 2020). In some plants, high cell densities (above OD_600_ = 1.0) induced wilting of infiltrated leaves (Kim *et al.* 2009) which we did not observe for sorghum. Additional factors improving transgene expression were incubation of the plants in the dark post-infiltration, and growing *Agrobacterium* densely on plates instead of in a liquid medium which aligns with the findings of Zhang *et al.* (2020). Using the *Agrobacterium* strain EHA105 instead of GV3101 did not result in higher expression. Kim *et al.* (2009) report that in Arabidopsis, EHA105 resulted in higher transgene expression than GV3101 when using a lower cell density (OD_600_ = 0.3) whereas GV3101 performed better at higher cell densities, in good accordance with our results achieved for sorghum.

With the changes made to the protocol, we managed to achieve a significant increase in transgene expression, but still observed substantial variability between experiments and in some cases did not detect any expression at all. Sharma *et al.* (2020) also reported low transformation efficiency with GFP alone, but not the same issues with autofluorescence. However, it is difficult to evaluate from their presented images if some of the signals could originate from the wounding response that looked quite distinct from the DHR2-GFP expression in our results. In the future, the use of other reporter genes may eliminate the problem of autofluorescence, such as GUS (Jefferson *et al.* 1987) or RUBY (He *et al.* 2020). Either way, for us as well as Sharma *et al.* (2020), the method is not nearly as robust as transient expression in, for example, *N. benthamiana*, and still requires further optimization. Furthermore, *N. benthamiana* is frequently used to characterize enzyme activities based on the detection of metabolites produced by the polypeptides encoded by the infiltrated genes (Eljounaidi and Lichman 2020). With the extremely low number of cells expressing the genes infiltrated in sorghum, the method is currently unlikely to be useful for enzyme characterization. Assembling biosynthetic pathways of multiple genes is also difficult if successful infiltration requires an OD of 2.0 for each gene. Improving the diffusion of *Agrobacterium* into the sorghum leaf is likely to improve most of the reported issues as also pointed out by Sharma *et al.* (2020). Furthermore, in our experiments transgene expression was more easily achieved in spring than in summer, indicating that similar to the situation for stable transformation of leaf whorls, plant growth conditions are crucial for successful transient gene expression. Hence, growing plants in a fully controlled environment could potentially improve transient transformation.

### Sub-cellular localization of *Sb*DHR2

Contrary to our expectations, DHR2 was mainly expressed in the cytosol and visibly diffusing into the nucleus when the expression was especially strong. From previous studies using protoplasts (Thayer and Conn 1981) and from predictions using TargetP 2.0 (Emanuelsson *et al.* 2007), we expected DHR2 to be localized in the chloroplasts. However, similar to agroinfiltration in *N. benthamiana (**Bally et al. 2018*), we only detected gene expression in epidermal cells. While *N. benthamiana* does have some chloroplasts in epidermal cells (Irieda and Takano 2021), sorghum, like most other plants, does not (Wurtele *et al.* 1982). We did, however, detect an aggregation of GFP in small granular structures which could be etioplasts, a type of plastids that can be found in the sorghum epidermis (Wurtele *et al.* 1982). Whereas chloroplasts can be detected using the signal resulting from chlorophyll fluorescence, it is almost impossible to detect etioplasts in living cells (Niwa *et al.* 1999). We, therefore, conclude that transient expression by agroinfiltration only has limited application for studies of sub-cellular localisation as chloroplast-targeted enzymes cannot be correctly localized.

## Conclusion

Both sorghum transformation methods tested in this study have potential but require further optimization. We successfully produced embryogenic callus from leaf whorls and expressed GFP-tagged sorghum proteins in sorghum leaves via agroinfiltration. However, both methods are currently neither robust nor simple and still need considerable optimization to be successfully used for sorghum research in temperate climates. We also demonstrated that agroinfiltration in sorghum leads to transgene expression confined to the epidermal cells. As they lack chloroplasts, this is something to be aware of when using this approach for sub-cellular localization studies.

## Supporting Information

The following additional information is available in the online version of this article –

Table S1. Media used for leaf whorl culture.

Protocol S2. Plasmid purification protocol.

Table S3. Primers used for plasmid construction.

Figure S4. Modified pCAMBIA1300.

Figure S5. Sequence of the *Zea mays* codon-optimized eGFP.

Figure S6. Expression of DHR2-GFP 4 DPI.

plad030_suppl_Supplementary_Supporting_Information

## Data Availability

All relevant data are included in the main text or supplementary information. The authors can be contacted if any further information is needed.

## Notes

**Phenome, Genome and Environment. Chief Editor:** Colleen Doherty
