# Supplementary material for: New methods for sorghum transformation in temperate climates
Source: AoB Plants. 2023 Jun 3;15(3):plad030. doi: 10.1093/aobpla/plad030 (PMC10308921; doi:10.1093/aobpla/plad030)
Supplement: plad030_suppl_Supplementary_Supporting_Information [file plad030_suppl_supplementary_supporting_information.pdf]

Supporting Information

Table S1. Media used for leaf whorl culture: All media were based on MS medium with Gamborg vitamins (44.4 g/L) with 8 g/L of phytoagar.

	**Callus Induction Medium (CIM)**				**Osmo-tic medium (OM)**	**Regeneration Medium (REM)**				**Rooting Medium (RM)**			
**Components**	**1**	**2**	**3**	**4**		**1**	**2**	**3**	**4**	**1**	**2**	**3**	**4**
**Sucrose [g/L]**	30	-	30	-	30	30	-	30	-	30	-	30	-
**Maltose [g/L]**	-	30	-	30		-	30	-	30	-	30	-	30
**L-proline [g/L]**	1	1	1	1		-	-	-	-	-	-	-	-
**L-asparagine [g/L]**	1	1	1	1		-	-	-	-	-	-	-	-
**Potassium dihydrogenphosphate (KH_2PO_4) [g/L]**	1	1	1	1		-	-	-	-	-	-	-	-
**D-Manitol [g/L]**					36.4								
**D-Sorbitol [g/L]**					36.4								
**2,4-Dichlorophenoxyacetic acid (2,4-D) [mg/L]**	1	1	1	1		-	-	-	-	-	-	-	-
***Copper Sulfate ($CuSO_4$) [µM]**	1	1	1	1		1	1	1	1	1	1	1	1
**6-Benzylaminopurine (BAP) [mg/L]**	-	-	-	-		1	1	1	1	-	-	-	-
**Indole-3-acetic acid (IAA) [mg/L]**	-	-	-	-		1	1	1	1	1	1	1	1
**Indole-3-butyric acid (IBA) [mg/L]**	-	-	-	-		-	-	-	-	1	1	1	1
**1-Naphthaleneacetic acid (NAA) [mg/L]**	-	-	-	-		-	-	-	-	1	1	1	1
**α-lipoic acid [mg/L]**	-	-	1	1		-	-	1	1	-	-	1	1
**pH**	5.7												

Protocol S2. Plasmid purification protocol

1: Centrifuge 1½-5 ml overnight culture 6 min at 4000 x g

2: Resuspend pellet in 100 µl TGE buffer (SOL I) + RNAse (1 µl/ml).

3: Lyse the bacteria in 200 µl 0.2M NaOH/ 1 % SDS freshly made (Dilute a 2M NaOH and a 10%SDS 10x). Invert the tube.

4: Add 150 µl Potassium acetate buffer (SOL III). Vortex, leave for 5 min on ice.

5: Centrifuge 10 min at 20000 x g

6: Transfer the supernatant to a new tube. Ad 800 µl 96 % EtOH, invert the tube.

7: Centrifuge 30 min at 20000 x g

8: Carefully pour off the supernatant. Wash pellet with 50 µl 70% EtOH

9: Centrifuge 5 - 10 min at 20000 x g

10: Remove the supernatant and air-dry pellet.

11: Dissolve the DNA in 20 – 50 µl H2O or 1x TE

Solutions: (Both autoclaved)

SOL I (TGE buffer)

50mM Glucose

25mM TRIS pH 8.0

10mM EDTA pH 8.0

Add RNAse A (1ul/ml)

SOL III

5M Potassium acetate in 60 ml H_2O

11.5 ml (100%) acetic acid=glacial acetic acid. (14,4ml 80%)

H_2O ad 100 ml (28.5 ml if glacial acetic; 25.6 if 80% acetic acid)

Table S3. Primers used for plasmid construction.

Name	*Sequence*
SbUbi-2_forward_2R	TAAGCACTGCAGCTCCCATCTCCCTTGCTTT
SbUbi-2_reverse_2R	TGCTTACTGCAGATTTGTGCGTGTCATCCTT
SbUbi-3_forward_2R	TAAGCACTGCAGCACCGCGAATGGATAAGAA
SbUbi-3_reverse_2R	TGCTTACTGCAGAAAGCCGAACACCAGGGA
pCAMBIA1300_gibson_fw	ATTTGGAGAGGACGACCTG
pCAMBIA1300_gibson_rev	GTCATAGCTGTTTCCTGTG
ZmUbi_gibson_fw	ACACAGGAAACAGCTATGACAATTCCTGCAGTGCAGCG
ZmUbi_gibson_rev	GCAGGTCGTCCTCTCCAAATCTGCAGAAGTAACACCAAACAAC
eGFP_USER_fw	GGCTTAAUGTAGCGGTGGAATGGTGA
eGFP_USER_rev	GTTTAAUCGGGGCTGAGGTTTAATTT
P19_USER_fw	GGCTTAAUCATTTGGAGAGGACAGCC
P19_USER_rev	GGTTTAAUCCCCTTACTCGCTTTCTTT
DHR2_USER_forward	GGTTTAAUCTAAGCTGGCGTAACAACTTTG
DHR2_USER_reverse	GGTTTAAUCTAAGCTGGCGTAACAACTTTG
attB DHR2.FOR	GGGGACAAGTTTGTACAAAAAAGCAGGCTTAATGGCTCCACTACTGCTTCATG
attB DHR2.REV	GGGGACCACTTTGTACAAGAAAGCTGGGTTAGCTGGCGTAACAACTTTGAT

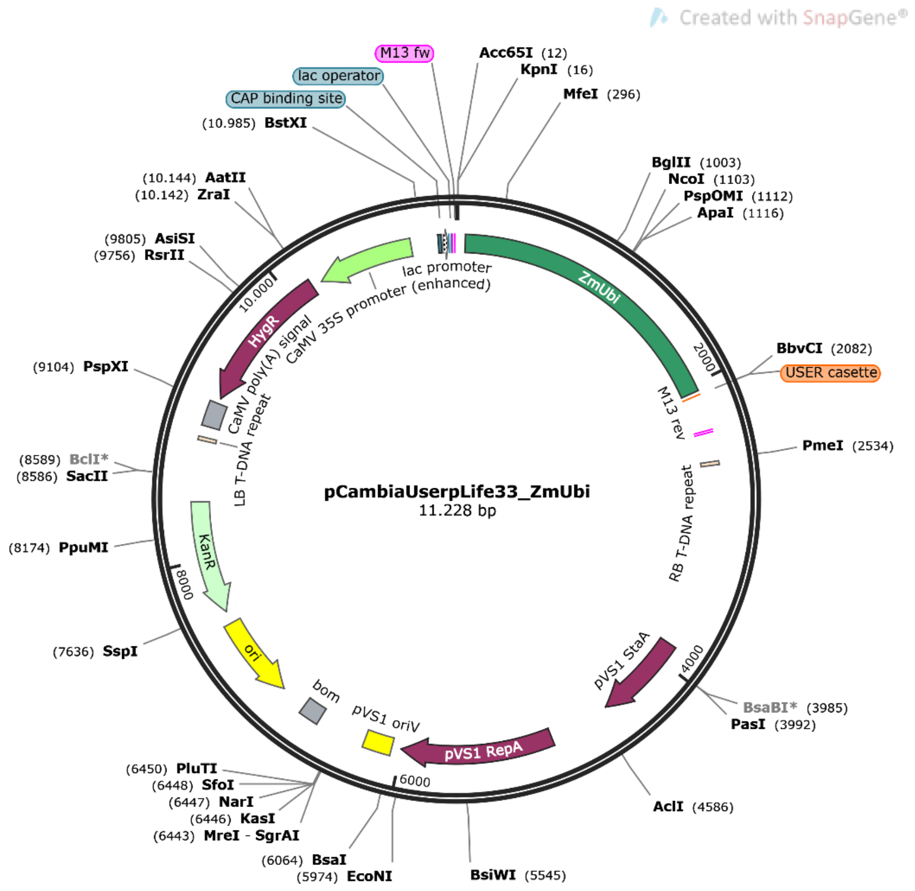

Figure S4. Modified pCAMBIA1300

pCAMBIA1300 was modified to contain promoters more suitable for sorghum transformation. The map shows a version of pCAMBIA1300 where the CaMV 35S promoter before the USER cassette has been replaced with a *Zm*Ubi. Two more versions of this vector were created where the original CaMV 35S promoter was replaced with *Sb*U6-2 and *Sb*U6-3 respectively. These plasmids were used to express maize optimised eGFP which was inserted via USER® Cloning (Nour-Eldin et al., 2006).

Figure S5. Sequence of the *Zea mays* codon optimised eGFP

```
ATGGTGTCCAAAGGTGAGGAGCTGTTCACAGGCGTCGTGCCGATTCTTGTGGAATTAGATGGAGACGT
CAATGGCCACAAATTTTCAGTTTCCGGAGAGGGGGAGGGCGACGCTACCTACGGCAAGCTTACTCTCA
AGTTCATATGCACAACGGGCAAACTCCCCGTTCCATGGCCAACGTTAGTAACTACCCTCACATATGGA
GTACAATGTTTTTCTCGTTATCCCGACCACATGAAGCAGCATGATTTCTTTAAGAGCGCCATGCCTGA
AGGGTATGTGCAGGAACGCACCATCTTCTTCAAAGATGACGGAAACTACAAAACAAGGGCTGAGGTGA
AGTTTGAGGGTGACACCCTGGTGAATCGGATTGAACTTAAAGGTATAGATTTTAAGGAGGACGGCAAC
ATCCTGGGGCACAAGCTAGAGTACAACTATAATTCTCACAATGTTTACATCATGGCAGACAAGCAAAA
GAACGGCATCAAGGTCAACTTCAAGATTCGACATAACATTGAAGATGGTTCAGTTCAATTGGCCGACC
ACTACCAGCAGAACACTCCGATAGGTGATGGGCCGGTGCTGCTGCCAGATAATCATTATCTTAGTACG
CAGAGCGCACTCTCGAAGGACCCTAATGAGAAGAGAGATCATATGGTCTTGTTGGAGTTCGTCACTGC
GGCGGGGATCACCCTAGGCATGGATGAACTCTACAAATGA
```

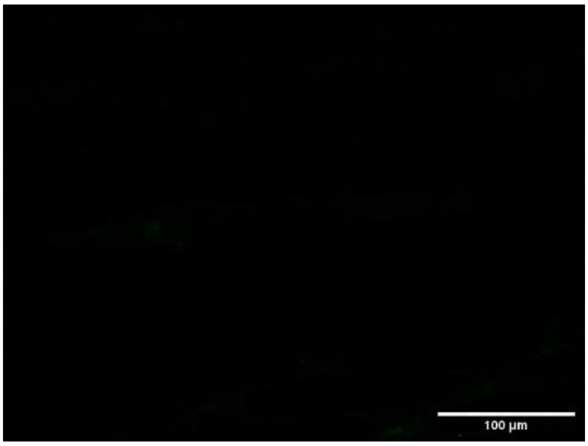

Figure S6. Expression of DHR2-GFP

The image was taken 4 DPI
